# Molecular detection of *Bartonella henselae* in 11 *Ixodes ricinus* ticks extracted from a single cat

**DOI:** 10.1186/s13071-017-2042-7

**Published:** 2017-03-13

**Authors:** Yvonne Regier, Wibke Ballhorn, Volkhard A. J. Kempf

**Affiliations:** University Hospital, Goethe-University, Institute for Medical Microbiology and Infection Control, Frankfurt am Main, Germany

**Keywords:** Vector-borne infections, Transmission, Zoonosis, *BadA*, *VirB*, Feline bartonellosis

## Abstract

**Background:**

*Bartonella henselae* is a highly prevalent, vector-borne pathogen. Transmission to humans and animals by ticks is discussed controversially. Here, we present a case report, where eleven *Ixodes ricinus* ticks all harbouring *B. henselae* DNA were removed from one single cat.

**Results:**

The first feeding tick was tested positive for *B. henselae* DNA. The cat was also found to be seropositive for anti-*B. henselae* IgG antibodies (titer 1:640). *Bartonella henselae* was not cultivatable from cat blood. Ten more feeding ticks removed 7 months later contained also *B. henselae* DNA. Sequence analysis of the 16SrDNA and the 16S-23S internal transcribed spacer (ITS) region revealed 100% sequence homology between all ticks. *Bartonella* adhesin A (*badA*) and VirB/VirD4 type IV secretion system (*virB*) DNA were also detected in all ticks.

**Conclusions:**

Our results indicate that cats may serve as a reservoir for adult ticks to acquire *B. henselae*. Whether this observation implies an increased threat for human and animal health needs to be resolved.

**Electronic supplementary material:**

The online version of this article (doi:10.1186/s13071-017-2042-7) contains supplementary material, which is available to authorized users.

## Background

*Bartonella henselae* is a Gram-negative, facultative intracellular, zoonotic pathogen [1]. In its reservoir host “cat” *B. henselae* causes a long lasting, intraerythrocytic, clinically asymptomatic bacteremia [2, 3]. However, cats may also develop diseases like endocarditis [4] and febrile illness [5]. Prevalence of anti-*Bartonella* antibodies in cat populations ranges from 0% in Norway [6] to 71% in Spain [7]. Immunocompetent humans can be infected with *B. henselae* by cat scratches or bites and may suffer thereafter from cat scratch disease [1], whereas immunocompromised individuals may develop vasoproliferative diseases (bacillary angiomatosis, peliosis hepatis [8, 9]). Among cats, *B. henselae* is transmitted *via* cat fleas by contamination of wounds with infected flea feces [10]. Other ectoparasites, e.g. ticks, are also suspect vectors for *B. henselae* [11]. Prevalence of *B. henselae* DNA in *Ixodes ricinus* varies from 0% in Finland [12] to 60% in the Netherlands [13]. Although vector competence of ticks for *B. henselae* has not been experimentally proven in vivo, an in vitro model employing an artificial feeding system successfully demonstrated transmission of *B. henselae* by ticks [14]. Furthermore, in a mouse infection model, the vector competence of ticks has been demonstrated for the murine pathogen *Bartonella birtlesii* [15]. However, discussions about the role of ticks as vectors for *B. henselae* are ongoing [16]. This case report describes the detection of *B. henselae* DNA in 11 ticks removed from one cat with an anti-*B. henselae* IgG titer of 1:640.

## Methods

### Sample drawing

Feeding ticks were removed from a 7-year-old, male, roaming Norwegian forest cat from Rastatt, Germany (48°51′N, 8°12′E) and stored at -20 °C in separate tubes containing ethanol. As the cat is a roaming cat with access to a rural area, tick contact occurs frequently. The first tick was removed in November 2015 and 10 more ticks were taken between March and June 2016 whenever a tick was detected by the owner. Ticks were identified using standard taxonomic keys (e.g. number of legs, shield, genital orifice [17]). For medical reasons and to exclude undiagnosed infections, cat serum was taken in December 2015 by venipuncture of the *vena cephalica* with a sterile 20 G needle. Blood was collected in a serum tube. After coagulation at room temperature for 40 min the tube was centrifuged for 10 min at 4000 rpm. Serum supernatant was stored at -20 °C.

Because of the elevated anti *B. henselae*-IgG titer from December 2015 and facing the detection of *B. henselae* DNA in all ticks taken from this cat in between, whole blood was taken for medical reasons in September 2016 for a detection attempt of *B. henselae* (by cultivation or PCR methods). To access the *vena cephalica*, the hair was clipped, the skin disinfected with 70% ethanol and the vein punctured with a sterile 20 G needle. Blood was collected in sterile tubes containing 10 μl of ethylendiaminetetraacetic acid disodium salt solution (Sigma-Aldrich, Steinheim, Germany) as anticoagulant.

### Quality control

The laboratories of the Institute for Medical Microbiology and Infection Control at the University Hospital of the Goethe University in Frankfurt (Germany) undergo a strict quality control management according to accredited standard operating procedures (laboratory accreditation according to ISO 15189:2007 standards; certificate number D-ML-13102-01-00, valid through January 25th, 2021). There was no increase of *Bartonella-*positive cases during this study; therefore, the possibility of DNA contamination from non-study sources is highly unlikely.

### DNA-extraction from ticks

Ticks were removed from their storage tubes with a sterile forceps, rinsed once in ethanol and twice in sterile water. After grinding each tick with a disposable sterile mortar and pestle, DNA was extracted with the QIAamp DNA Mini kit (Qiagen, Hilden, Germany) according to the manufacturer’s instructions. To prevent DNA cross-contamination, each tick was processed individually by using new forceps, tubes and mortars and pestles. Extraction procedure was verified using specific pathogen-free ticks (Insect Services, Berlin, Germany) in which *Bartonella* spp. was not detected (data not shown).

### Culturing of blood samples

A hundred μl of blood was immediately plated onto Columbia blood agar (BD, Heidelberg, Germany), chocolate agar plates (Oxoid, Wesel, Germany) moreover, 100 μl were suspended in fresh, quality-controlled *Bartonella*-liquid-medium [18]. Due to the limited amount of cat blood, no higher volumes were available for the inoculation of liquid cultures. Bacterial cultivation was performed for 8 weeks at 37 °C with 5% CO_2_ and 95% humidity. The residual blood was frozen at -80 °C overnight, thawed at 37 °C and plated as described. Once per week (over a total period of 8 weeks), 100 μl of the liquid cultures were plated onto CBA plates and incubated as described above. PCR analysis from liquid cultures medium was done after 18 days and after 60 days of incubation, respectively.

### DNA extraction from blood and liquid cultures

DNA from the cat’s blood and the liquid cultures was extracted using the DNeasy Blood and Tissue Kit (Qiagen, Hilden, Germany) according to manufacturer’s instructions.

### Polymerase chain reaction

A nested PCR for the detection of the *Bartonella* 16S ribosomal DNA (rDNA) was performed as previously described using the Taq DNA Polymerase-Kit (Invitrogen, Schwerte, Germany) [19, 20]. Furthermore, a PCR detecting the 16S-23S-rRNA internal transcribed spacer (ITS) region of *Bartonella* was conducted using the Platinum Taq Polymerase-Kit (Invitrogen, Schwerte, Germany) to distinguish *Bartonella* species [21]. PCR detection of the *Bartonella* pathogenicity factors *badA* and *virB* was conducted with the Pwo SuperYield DNA Polymerase (Roche, Mannheim, Germany). All PCR primers and annealing temperatures are listed in Table 1. Positive and a negative (water) control were always included. DNA was amplified in a Biometra T3000 thermocycler. Products were separated on an agarose gel, ethidiumbromide-stained and visualized under UV light.

**Table 1:** Primer designation, sequences and annealing temperatures of the conducted PCRs used for the detection of *Bartonella* spp. from *Ixodes ricinus* ticks

Target	Primer designation	Sequence (5′–3′)	Length (bp)	Annealing temperature (°C)	Reference
*Bartonella* spp. 16S rDNA (first round)	A-proteo primer	AGAGTTTGATC(AC)TGGCTCAGA	1,210	62 °C (1 min)	[19]
	r-Alpha-sh primer	GTAGCACGTGTGTAGCCCA			
*Bartonella* spp. 16S rDNA (nested PCR)	Bart	CACTCTTTTAGAGTGAGCGGCAA	990	65 °C (1 min)	[19]
	r-BH	CCCCCTAGAGTGCCCAACCA			
*Bartonella* 16S-23S ITS	325 s	CTTCAGATGATGATCCCAAGCCTTCTGGCG	489	68 °C (15 s)	[21]
	1100as	GAACCGACGACCCCCTGCTTGCAAAGCA			
*Bad A* head region	badAf8	TCGAATCTTGCGCTTACAGGAGC	325	59 °C (30 s)	Present study
	BadA_head_reverse	CACCGTCAGTCGACTTCCCT			
*Vir B7* of the *VirB/D4*-type-IV-secretion-system	VirB7_for	GCTGGAAAACGAAAAAGCAA	103	55 °C (30 s)	Present study
	VirB7_rev	ACGCGCAATCTCCATAGTGT			

### Sequencing and alignment

16S rDNA and ITS PCR products were sequenced (GATC, Konstanz, Germany) with both, forward and reverse primers. Sequences were checked using Chromas software (Technelysium, Version 2.6, South Brisbane, Australia), aligned and compared to *B. henselae* strain BM1374165 (GenBank: HG969191.1) using Clone Manager Professional Suite version 8 (Scientific and Educational Software, Denver, USA).

### Immunofluorescence assay

Indirect immunofluorescence assay (IIFA) was performed using the *Bartonella henselae/ Bartonella quintana* (IgG) kit (Euroimmun, Lübeck, Germany) with some modifications. Serum dilution series from 1:20 to 1:2,560 were screened for *Bartonella* cat IgG antibodies. A 1:100 dilution of Alexa Fluor 488-conjugated AffiniPure Goat Anti-Cat IgG (Jackson ImmunoResearch laboratories, West Grove, USA) was used as secondary antibody. The test was evaluated as positive when specific fluorescence was detected at a titer of ≥ 1:64 [22].

## Results

**Fig. 1:**
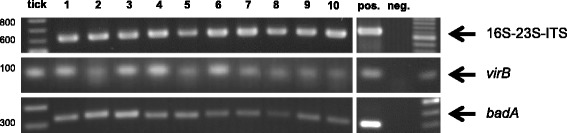
PCR products from the 16S-23S-ITS-, *virB*- and the *badA*-PCR. Results conducted from DNA of the ten ticks collected from March to June 2016. Positive control: *Bartonella henselae* Houston ATCC 49882, Negative control: distilled water (representative example)

All ticks were female, adult, half- to fully-engorged and were identified as *Ixodes ricinus*. One feeding tick, which was removed from the cat in November 2015, was tested positive for *B. henselae* in 16S rDNA PCR analysis. In December 2015, the same cat’s serum was screened for anti-*Bartonella* IgG using an indirect immunofluorescence assay (IIFA), revealing a titer of 1:640 (cut-off: 1:64 [22]). No serum was available from September 2016 for determination of anti-*Bartonella* IgG. Subsequently, between March and June 2016, ten more feeding ticks were removed from the same cat and tested positive for *B. henselae via* PCR amplification of 16S rDNA, 16S-23S-ITS, *virB*- and *badA* (Fig. 1). PCR products of the 16S rDNA and ITS were sequenced and alignment showed 100% sequence homology each between all 11 ticks and also to *B. henselae* strain BM1374165 (Positions: ITS: 1483445-1483766, 16S rDNA: 1484693-1485539) (GenBank: HG969191.1) (see Additional file 1: Figure S1 and Additional file 2: Figure S2). No *Bartonella* DNA was detected in the cat’s blood taken in September 2016 and in six different cultural attempts from continuously incubated liquid cultures. Blood plated on solid media and suspended in liquid media remained negative over an incubation period of 8 weeks.

## Discussion

*Bartonella* spp. are highly prevalent and are found in ticks all over the world [11]. The sampling site of this study (Rastatt, Germany) is only 35 km away from Lembach, France, where a previous study showed highest prevalence of *B. henselae* in ticks in Europe (38% in nymphs and 12% in adult ticks) indicating that there might be an increased prevalence of *Bartonella*-infected ticks in this area [20]. Cats, especially roaming cats, have a high risk of being infested with ticks and other ectoparasites such as fleas which can harbor or transmit *Bartonella* spp. and other infectious agents such as *Anaplasma* spp. [10, 11, 20, 23]. In this case, due to a strict ectoparasite control, no fleas were detected on the cat.

*Bartonella* adhesin A (BadA) mediates adhesion of *Bartonella* spp. to endothelial cells and the extracellular matrix proteins [24]. The VirB/VirD4 type IV secretion system (VirB) translocates *Bartonella* effector proteins (Beps) into endothelial host cells, e.g. inhibiting apoptosis, and inducing a proinflammatory phenotype, which is responsible for the chronicity of the infection and involved in the cell invasion process of *Bartonella* spp. [25, 26]. We detected the DNA of *badA* and *virB* in all eleven ticks analyzed herein. This indicates that these two *Bartonella* pathogenicity factors are at least present in the genomes of the detected *B. henselae* and might give clues for estimating the extent of the potential health threat for humans and animals infected with those bacteria.

The cat examined in this study was tested seropositive for *Bartonella* spp. in December 2015 (titer 1:640). Clearance of bacteremia due to presence of anti-*Bartonella* IgG antibodies could be a possible reason for the negative results of the cultures and PCR from the cat’s blood drawn in September 2016. When infected with the murine pathogen *B. grahamii*, immunocompromised mice cleared the resulting intraerythrocytic bacteremia after administration of IgG antibodies obtained from immunocompetent mice [27]. In another study, cats were shown to clear *Bartonella* bacteremia within 1–8 months after being experimentally inoculated with *B. henselae* subcutaneously [28]. As the cat described herein had a significant serum IgG titer against *B. henselae*, this might indicate that it had already cleared the bacteremia thereby explaining the several negative PCR and cultivation attempts from the peripheral blood. Another possible reason for the lack of viable *Bartonella* or *Bartonella* DNA in the blood could be that bacteremia was below the detection limit at the time of blood withdrawal. This hypothesis is supported by a study in which alternating periods of bacteremia were observed in a naturally infected cat over a period of 24 months, with periods of high bacteremia and periods where blood cultures were found to be negative [28].

Furthermore, using a *B. tribocorum* rat infection model, skin dendritic cells have been described to act as vehicles from the primary site of infection (skin) towards the blood stream depending on the function of the *Bartonella* effector protein E (BepE) [29]. Accordingly, the DNA of all ticks analyzed in our case report contained DNA encoding for the VirB/D4 secretion system (injecting BepE into eukaryotic cells) showing that this machinery is present in our *B. henselae*-genome. This suggests that *B. henselae* might in fact preferentially occupy the dermal niche of cats to enhance vector acquisition by ticks.

The origin of *B. henselae* DNA found in the adult *I. ricinus* ticks of this survey remains speculative. Ticks undergo three life stages as larvae, nymphs and adult ticks. Each life stage takes one single blood meal, providing three possibilities of being infected with *B. henselae* or other infectious agents. We consider infection of the adult ticks examined in this study with *B. henselae* at an earlier life stage unlikely, since sequencing of PCR products showed 100% sequence homologies for each analyzed (gene) sequence. This points to the possibility, that all ticks became infected by the same host, the cat; however 16S and 16S-23S-ITS genes are known to be highly conserved among *B. henselae* isolates. Whether the *B. henselae* found in ticks were viable remains unclear since we prioritized DNA extraction and therefore did not attempt cultivation of bacteria from ticks.

Engorged, infected, adult hard ticks pose a low risk to human or animal health since they usually only have a single blood meal. However, infection of humans and animals might be possible, since transstadial transmission of *Bartonella* spp. in *I. ricinus* occurs when larvae or nymphs feed on infected hosts and progress into the next life stage (larvae to nymph or nymph to adult tick) [14]. Transovarial transmission of *Bartonella* spp. by ticks is not yet fully resolved. Unengorged larvae were found to harbor *Bartonella* DNA. Here, vertical transmission of *Bartonella* spp. might be the explanation for presence of bacteria, if those larvae did not have an undetected blood meal [30]. Furthermore, *B. henselae* was detected in eggs from female ticks which were fed with infected blood but no *Bartonella* DNA was amplified from the larvae eclosed from those eggs [14].

## Conclusions

In summary, this case study shows prevalence of *Bartonella* spp. in a cat and their ectoparasites (ticks). The circulation of *B. henselae* is maintained by ectoparasites and animals. A lot more research has to be done to elucidate the role of ticks in the transmission of *B. henselae* to estimate the risk of infection to humans and pets.

## Additional files

Additional file 1: Figure S1. Alignment 16S rDNA. (DOCX 14 kb)
Additional file 2: Figure S2. Alignment 16S-23S-ITS. (DOCX 14 kb)

## Abbreviations

*badA*: *Bartonella* adhesin A gene
Bep: *Bartonella* effector protein
DNA: Deoxyribonucleic acid
IgG: Immunoglobulin g
IIFA: Indirect immunofluorescence assay
ITS: Internal transcribed spacer
PCR: Polymerase chain reaction
*virB*: VirB/VirD4 type IV secretion system gene
