# Supplementary material for: Molecular detection of Bartonella henselae in 11 Ixodes ricinus ticks extracted from a single cat
Source: Parasit Vectors. 2017 Mar 13;10:105. doi: 10.1186/s13071-017-2042-7 (PMC5346845; doi:10.1186/s13071-017-2042-7)
Supplement: Additional file 1: Figure S1. — Alignment 16S rDNA. (DOCX 14 kb) [file 13071_2017_2042_MOESM1_ESM.docx]

**Regier *et al.*, 2017: Molecular detection of *Bartonella henselae* in 11 *Ixodes ricinus* ticks extracted from a single cat**

**Additional file 1: Figure S1.** Alignment 16S rDNA

Reference molecule: B. henselae, strain BM1374165, GenBank: HG969191.1 Region 1484693 to 1485539

B. henselae 1 tacggaataacacagagaaatttgtgctaataccgtatac

16S tick 1 1 tacggaataacacagagaaatttgtgctaataccgtatac

16S tick 2 1 tacggaataacacagagaaatttgtgctaataccgtatac

16S tick 3 1 tacggaataacacagagaaatttgtgctaataccgtatac

16S tick 4 1 tacggaataacacagagaaatttgtgctaataccgtatac

16S tick 5 1 tacggaataacacagagaaatttgtgctaataccgtatac

16S tick 6 1 tacggaataacacagagaaatttgtgctaataccgtatac

16S tick 7 1 tacggaataacacagagaaatttgtgctaataccgtatac

16S tick 8 1 tacggaataacacagagaaatttgtgctaataccgtatac

16S tick 9 1 tacggaataacacagagaaatttgtgctaataccgtatac

16S tick 10 1 tacggaataacacagagaaatttgtgctaataccgtatac

16S tick 11 1 tacggaataacacagagaaatttgtgctaataccgtatac

B. henselae 41 gtcctatttggagaaagatttatcggagatggatgagccc

16S tick 1 41 gtcctatttggagaaagatttatcggagatggatgagccc

16S tick 2 41 gtcctatttggagaaagatttatcggagatggatgagccc

16S tick 3 41 gtcctatttggagaaagatttatcggagatggatgagccc

16S tick 4 41 gtcctatttggagaaagatttatcggagatggatgagccc

16S tick 5 41 gtcctatttggagaaagatttatcggagatggatgagccc

16S tick 6 41 gtcctatttggagaaagatttatcggagatggatgagccc

16S tick 7 41 gtcctatttggagaaagatttatcggagatggatgagccc

16S tick 8 41 gtcctatttggagaaagatttatcggagatggatgagccc

16S tick 9 41 gtcctatttggagaaagatttatcggagatggatgagccc

16S tick 10 41 gtcctatttggagaaagatttatcggagatggatgagccc

16S tick 11 41 gtcctatttggagaaagatttatcggagatggatgagccc

B. henselae 81 gcgttggattagctagttggtgaggtaacggctcaccaag

16S tick 1 81 gcgttggattagctagttggtgaggtaacggctcaccaag

16S tick 2 81 gcgttggattagctagttggtgaggtaacggctcaccaag

16S tick 3 81 gcgttggattagctagttggtgaggtaacggctcaccaag

16S tick 4 81 gcgttggattagctagttggtgaggtaacggctcaccaag

16S tick 5 81 gcgttggattagctagttggtgaggtaacggctcaccaag

16S tick 6 81 gcgttggattagctagttggtgaggtaacggctcaccaag

16S tick 7 81 gcgttggattagctagttggtgaggtaacggctcaccaag

16S tick 8 81 gcgttggattagctagttggtgaggtaacggctcaccaag

16S tick 9 81 gcgttggattagctagttggtgaggtaacggctcaccaag

16S tick 10 81 gcgttggattagctagttggtgaggtaacggctcaccaag

16S tick 11 81 gcgttggattagctagttggtgaggtaacggctcaccaag

B. henselae 121 gcgacgatccatagctggtctgagaggatgatcagccaca

16S tick 1 121 gcgacgatccatagctggtctgagaggatgatcagccaca

16S tick 2 121 gcgacgatccatagctggtctgagaggatgatcagccaca

16S tick 3 121 gcgacgatccatagctggtctgagaggatgatcagccaca

16S tick 4 121 gcgacgatccatagctggtctgagaggatgatcagccaca

16S tick 5 121 gcgacgatccatagctggtctgagaggatgatcagccaca

16S tick 6 121 gcgacgatccatagctggtctgagaggatgatcagccaca

16S tick 7 121 gcgacgatccatagctggtctgagaggatgatcagccaca

16S tick 8 121 gcgacgatccatagctggtctgagaggatgatcagccaca

16S tick 9 121 gcgacgatccatagctggtctgagaggatgatcagccaca

16S tick 10 121 gcgacgatccatagctggtctgagaggatgatcagccaca

16S tick 11 121 gcgacgatccatagctggtctgagaggatgatcagccaca

B. henselae 161 ctgggactgagacacggcccagactcctacgggaggcagc

16S tick 1 161 ctgggactgagacacggcccagactcctacgggaggcagc

16S tick 2 161 ctgggactgagacacggcccagactcctacgggaggcagc

16S tick 3 161 ctgggactgagacacggcccagactcctacgggaggcagc

16S tick 4 161 ctgggactgagacacggcccagactcctacgggaggcagc

16S tick 5 161 ctgggactgagacacggcccagactcctacgggaggcagc

16S tick 6 161 ctgggactgagacacggcccagactcctacgggaggcagc

16S tick 7 161 ctgggactgagacacggcccagactcctacgggaggcagc

16S tick 8 161 ctgggactgagacacggcccagactcctacgggaggcagc

16S tick 9 161 ctgggactgagacacggcccagactcctacgggaggcagc

16S tick 10 161 ctgggactgagacacggcccagactcctacgggaggcagc

16S tick 11 161 ctgggactgagacacggcccagactcctacgggaggcagc

B. henselae 201 agtggggaatattggacaatgggggcaaccctgatccagc

16S tick 1 201 agtggggaatattggacaatgggggcaaccctgatccagc

16S tick 2 201 agtggggaatattggacaatgggggcaaccctgatccagc

16S tick 3 201 agtggggaatattggacaatgggggcaaccctgatccagc

16S tick 4 201 agtggggaatattggacaatgggggcaaccctgatccagc

16S tick 5 201 agtggggaatattggacaatgggggcaaccctgatccagc

16S tick 6 201 agtggggaatattggacaatgggggcaaccctgatccagc

16S tick 7 201 agtggggaatattggacaatgggggcaaccctgatccagc

16S tick 8 201 agtggggaatattggacaatgggggcaaccctgatccagc

16S tick 9 201 agtggggaatattggacaatgggggcaaccctgatccagc

16S tick 10 201 agtggggaatattggacaatgggggcaaccctgatccagc

16S tick 11 201 agtggggaatattggacaatgggggcaaccctgatccagc

B. henselae 241 catgccgcgtgagtgatgaaggccctagggttgtaaagct

16S tick 1 241 catgccgcgtgagtgatgaaggccctagggttgtaaagct

16S tick 2 241 catgccgcgtgagtgatgaaggccctagggttgtaaagct

16S tick 3 241 catgccgcgtgagtgatgaaggccctagggttgtaaagct

16S tick 4 241 catgccgcgtgagtgatgaaggccctagggttgtaaagct

16S tick 5 241 catgccgcgtgagtgatgaaggccctagggttgtaaagct

16S tick 6 241 catgccgcgtgagtgatgaaggccctagggttgtaaagct

16S tick 7 241 catgccgcgtgagtgatgaaggccctagggttgtaaagct

16S tick 8 241 catgccgcgtgagtgatgaaggccctagggttgtaaagct

16S tick 9 241 catgccgcgtgagtgatgaaggccctagggttgtaaagct

16S tick 10 241 catgccgcgtgagtgatgaaggccctagggttgtaaagct

16S tick 11 241 catgccgcgtgagtgatgaaggccctagggttgtaaagct

B. henselae 281 ctttcaccggtgaagataatgacggtaaccggagaagaag

16S tick 1 281 ctttcaccggtgaagataatgacggtaaccggagaagaag

16S tick 2 281 ctttcaccggtgaagataatgacggtaaccggagaagaag

16S tick 3 281 ctttcaccggtgaagataatgacggtaaccggagaagaag

16S tick 4 281 ctttcaccggtgaagataatgacggtaaccggagaagaag

16S tick 5 281 ctttcaccggtgaagataatgacggtaaccggagaagaag

16S tick 6 281 ctttcaccggtgaagataatgacggtaaccggagaagaag

16S tick 7 281 ctttcaccggtgaagataatgacggtaaccggagaagaag

16S tick 8 281 ctttcaccggtgaagataatgacggtaaccggagaagaag

16S tick 9 281 ctttcaccggtgaagataatgacggtaaccggagaagaag

16S tick 10 281 ctttcaccggtgaagataatgacggtaaccggagaagaag

16S tick 11 281 ctttcaccggtgaagataatgacggtaaccggagaagaag

B. henselae 321 ccccggctaacttcgtgccagcagccgcggtaatacgaag

16S tick 1 321 ccccggctaacttcgtgccagcagccgcggtaatacgaag

16S tick 2 321 ccccggctaacttcgtgccagcagccgcggtaatacgaag

16S tick 3 321 ccccggctaacttcgtgccagcagccgcggtaatacgaag

16S tick 4 321 ccccggctaacttcgtgccagcagccgcggtaatacgaag

16S tick 5 321 ccccggctaacttcgtgccagcagccgcggtaatacgaag

16S tick 6 321 ccccggctaacttcgtgccagcagccgcggtaatacgaag

16S tick 7 321 ccccggctaacttcgtgccagcagccgcggtaatacgaag

16S tick 8 321 ccccggctaacttcgtgccagcagccgcggtaatacgaag

16S tick 9 321 ccccggctaacttcgtgccagcagccgcggtaatacgaag

16S tick 10 321 ccccggctaacttcgtgccagcagccgcggtaatacgaag

16S tick 11 321 ccccggctaacttcgtgccagcagccgcggtaatacgaag

B. henselae 361 ggggctagcgttgttcggatttactgggcgtaaagcgcat

16S tick 1 361 ggggctagcgttgttcggatttactgggcgtaaagcgcat

16S tick 2 361 ggggctagcgttgttcggatttactgggcgtaaagcgcat

16S tick 3 361 ggggctagcgttgttcggatttactgggcgtaaagcgcat

16S tick 4 361 ggggctagcgttgttcggatttactgggcgtaaagcgcat

16S tick 5 361 ggggctagcgttgttcggatttactgggcgtaaagcgcat

16S tick 6 361 ggggctagcgttgttcggatttactgggcgtaaagcgcat

16S tick 7 361 ggggctagcgttgttcggatttactgggcgtaaagcgcat

16S tick 8 361 ggggctagcgttgttcggatttactgggcgtaaagcgcat

16S tick 9 361 ggggctagcgttgttcggatttactgggcgtaaagcgcat

16S tick 10 361 ggggctagcgttgttcggatttactgggcgtaaagcgcat

16S tick 11 361 ggggctagcgttgttcggatttactgggcgtaaagcgcat

B. henselae 401 gtaggcggatatttaagtcagaggtgaaatcccagggctc

16S tick 1 401 gtaggcggatatttaagtcagaggtgaaatcccagggctc

16S tick 2 401 gtaggcggatatttaagtcagaggtgaaatcccagggctc

16S tick 3 401 gtaggcggatatttaagtcagaggtgaaatcccagggctc

16S tick 4 401 gtaggcggatatttaagtcagaggtgaaatcccagggctc

16S tick 5 401 gtaggcggatatttaagtcagaggtgaaatcccagggctc

16S tick 6 401 gtaggcggatatttaagtcagaggtgaaatcccagggctc

16S tick 7 401 gtaggcggatatttaagtcagaggtgaaatcccagggctc

16S tick 8 401 gtaggcggatatttaagtcagaggtgaaatcccagggctc

16S tick 9 401 gtaggcggatatttaagtcagaggtgaaatcccagggctc

16S tick 10 401 gtaggcggatatttaagtcagaggtgaaatcccagggctc

16S tick 11 401 gtaggcggatatttaagtcagaggtgaaatcccagggctc

B. henselae 441 aaccctggaactgcctttgatactgggtatcttgagtgtg

16S tick 1 441 aaccctggaactgcctttgatactgggtatcttgagtgtg

16S tick 2 441 aaccctggaactgcctttgatactgggtatcttgagtgtg

16S tick 3 441 aaccctggaactgcctttgatactgggtatcttgagtgtg

16S tick 4 441 aaccctggaactgcctttgatactgggtatcttgagtgtg

16S tick 5 441 aaccctggaactgcctttgatactgggtatcttgagtgtg

16S tick 6 441 aaccctggaactgcctttgatactgggtatcttgagtgtg

16S tick 7 441 aaccctggaactgcctttgatactgggtatcttgagtgtg

16S tick 8 441 aaccctggaactgcctttgatactgggtatcttgagtgtg

16S tick 9 441 aaccctggaactgcctttgatactgggtatcttgagtgtg

16S tick 10 441 aaccctggaactgcctttgatactgggtatcttgagtgtg

16S tick 11 441 aaccctggaactgcctttgatactgggtatcttgagtgtg

B. henselae 481 gaagaggtgagtggaattccgagtgtagaggtaaaattcg

16S tick 1 481 gaagaggtgagtggaattccgagtgtagaggtaaaattcg

16S tick 2 481 gaagaggtgagtggaattccgagtgtagaggtaaaattcg

16S tick 3 481 gaagaggtgagtggaattccgagtgtagaggtaaaattcg

16S tick 4 481 gaagaggtgagtggaattccgagtgtagaggtaaaattcg

16S tick 5 481 gaagaggtgagtggaattccgagtgtagaggtaaaattcg

16S tick 6 481 gaagaggtgagtggaattccgagtgtagaggtaaaattcg

16S tick 7 481 gaagaggtgagtggaattccgagtgtagaggtaaaattcg

16S tick 8 481 gaagaggtgagtggaattccgagtgtagaggtaaaattcg

16S tick 9 481 gaagaggtgagtggaattccgagtgtagaggtaaaattcg

16S tick 10 481 gaagaggtgagtggaattccgagtgtagaggtaaaattcg

16S tick 11 481 gaagaggtgagtggaattccgagtgtagaggtaaaattcg

B. henselae 521 tagatattcggaggaacaccagtggcgaaggcggctcact

16S tick 1 521 tagatattcggaggaacaccagtggcgaaggcggctcact

16S tick 2 521 tagatattcggaggaacaccagtggcgaaggcggctcact

16S tick 3 521 tagatattcggaggaacaccagtggcgaaggcggctcact

16S tick 4 521 tagatattcggaggaacaccagtggcgaaggcggctcact

16S tick 5 521 tagatattcggaggaacaccagtggcgaaggcggctcact

16S tick 6 521 tagatattcggaggaacaccagtggcgaaggcggctcact

16S tick 7 521 tagatattcggaggaacaccagtggcgaaggcggctcact

16S tick 8 521 tagatattcggaggaacaccagtggcgaaggcggctcact

16S tick 9 521 tagatattcggaggaacaccagtggcgaaggcggctcact

16S tick 10 521 tagatattcggaggaacaccagtggcgaaggcggctcact

16S tick 11 521 tagatattcggaggaacaccagtggcgaaggcggctcact

B. henselae 561 ggtccattactgacgctgaggtgcgaaagcgtggggagca

16S tick 1 561 ggtccattactgacgctgaggtgcgaaagcgtggggagca

16S tick 2 561 ggtccattactgacgctgaggtgcgaaagcgtggggagca

16S tick 3 561 ggtccattactgacgctgaggtgcgaaagcgtggggagca

16S tick 4 561 ggtccattactgacgctgaggtgcgaaagcgtggggagca

16S tick 5 561 ggtccattactgacgctgaggtgcgaaagcgtggggagca

16S tick 6 561 ggtccattactgacgctgaggtgcgaaagcgtggggagca

16S tick 7 561 ggtccattactgacgctgaggtgcgaaagcgtggggagca

16S tick 8 561 ggtccattactgacgctgaggtgcgaaagcgtggggagca

16S tick 9 561 ggtccattactgacgctgaggtgcgaaagcgtggggagca

16S tick 10 561 ggtccattactgacgctgaggtgcgaaagcgtggggagca

16S tick 11 561 ggtccattactgacgctgaggtgcgaaagcgtggggagca

B. henselae 601 aacaggattagataccctggtagtccacgccgtaaacgat

16S tick 1 601 aacaggattagataccctggtagtccacgccgtaaacgat

16S tick 2 601 aacaggattagataccctggtagtccacgccgtaaacgat

16S tick 3 601 aacaggattagataccctggtagtccacgccgtaaacgat

16S tick 4 601 aacaggattagataccctggtagtccacgccgtaaacgat

16S tick 5 601 aacaggattagataccctggtagtccacgccgtaaacgat

16S tick 6 601 aacaggattagataccctggtagtccacgccgtaaacgat

16S tick 7 601 aacaggattagataccctggtagtccacgccgtaaacgat

16S tick 8 601 aacaggattagataccctggtagtccacgccgtaaacgat

16S tick 9 601 aacaggattagataccctggtagtccacgccgtaaacgat

16S tick 10 601 aacaggattagataccctggtagtccacgccgtaaacgat

16S tick 11 601 aacaggattagataccctggtagtccacgccgtaaacgat

B. henselae 641 gaatgttagccgtcgggcggtttactgctcggtggcgcag

16S tick 1 641 gaatgttagccgtcgggcggtttactgctcggtggcgcag

16S tick 2 641 gaatgttagccgtcgggcggtttactgctcggtggcgcag

16S tick 3 641 gaatgttagccgtcgggcggtttactgctcggtggcgcag

16S tick 4 641 gaatgttagccgtcgggcggtttactgctcggtggcgcag

16S tick 5 641 gaatgttagccgtcgggcggtttactgctcggtggcgcag

16S tick 6 641 gaatgttagccgtcgggcggtttactgctcggtggcgcag

16S tick 7 641 gaatgttagccgtcgggcggtttactgctcggtggcgcag

16S tick 8 641 gaatgttagccgtcgggcggtttactgctcggtggcgcag

16S tick 9 641 gaatgttagccgtcgggcggtttactgctcggtggcgcag

16S tick 10 641 gaatgttagccgtcgggcggtttactgctcggtggcgcag

16S tick 11 641 gaatgttagccgtcgggcggtttactgctcggtggcgcag

B. henselae 681 ctaacgcgttaaacattccgcctggggagtacggtcgcaa

16S tick 1 681 ctaacgcgttaaacattccgcctggggagtacggtcgcaa

16S tick 2 681 ctaacgcgttaaacattccgcctggggagtacggtcgcaa

16S tick 3 681 ctaacgcgttaaacattccgcctggggagtacggtcgcaa

16S tick 4 681 ctaacgcgttaaacattccgcctggggagtacggtcgcaa

16S tick 5 681 ctaacgcgttaaacattccgcctggggagtacggtcgcaa

16S tick 6 681 ctaacgcgttaaacattccgcctggggagtacggtcgcaa

16S tick 7 681 ctaacgcgttaaacattccgcctggggagtacggtcgcaa

16S tick 8 681 ctaacgcgttaaacattccgcctggggagtacggtcgcaa

16S tick 9 681 ctaacgcgttaaacattccgcctggggagtacggtcgcaa

16S tick 10 681 ctaacgcgttaaacattccgcctggggagtacggtcgcaa

16S tick 11 681 ctaacgcgttaaacattccgcctggggagtacggtcgcaa

B. henselae 721 gattaaaactcaaaggaattgacgggggcccgcacaagcg

16S tick 1 721 gattaaaactcaaaggaattgacgggggcccgcacaagcg

16S tick 2 721 gattaaaactcaaaggaattgacgggggcccgcacaagcg

16S tick 3 721 gattaaaactcaaaggaattgacgggggcccgcacaagcg

16S tick 4 721 gattaaaactcaaaggaattgacgggggcccgcacaagcg

16S tick 5 721 gattaaaactcaaaggaattgacgggggcccgcacaagcg

16S tick 6 721 gattaaaactcaaaggaattgacgggggcccgcacaagcg

16S tick 7 721 gattaaaactcaaaggaattgacgggggcccgcacaagcg

16S tick 8 721 gattaaaactcaaaggaattgacgggggcccgcacaagcg

16S tick 9 721 gattaaaactcaaaggaattgacgggggcccgcacaagcg

16S tick 10 721 gattaaaactcaaaggaattgacgggggcccgcacaagcg

16S tick 11 721 gattaaaactcaaaggaattgacgggggcccgcacaagcg

B. henselae 761 gtggagcatgtggtttaattcgaagcaacgcgcagaacct

16S tick 1 761 gtggagcatgtggtttaattcgaagcaacgcgcagaacct

16S tick 2 761 gtggagcatgtggtttaattcgaagcaacgcgcagaacct

16S tick 3 761 gtggagcatgtggtttaattcgaagcaacgcgcagaacct

16S tick 4 761 gtggagcatgtggtttaattcgaagcaacgcgcagaacct

16S tick 5 761 gtggagcatgtggtttaattcgaagcaacgcgcagaacct

16S tick 6 761 gtggagcatgtggtttaattcgaagcaacgcgcagaacct

16S tick 7 761 gtggagcatgtggtttaattcgaagcaacgcgcagaacct

16S tick 8 761 gtggagcatgtggtttaattcgaagcaacgcgcagaacct

16S tick 9 761 gtggagcatgtggtttaattcgaagcaacgcgcagaacct

16S tick 10 761 gtggagcatgtggtttaattcgaagcaacgcgcagaacct

16S tick 11 761 gtggagcatgtggtttaattcgaagcaacgcgcagaacct

B. henselae 801 taccagcccttgacatcccgatcgcggaaggtggagacac

16S tick 1 801 taccagcccttgacatcccgatcgcggaaggtggagacac

16S tick 2 801 taccagcccttgacatcccgatcgcggaaggtggagacac

16S tick 3 801 taccagcccttgacatcccgatcgcggaaggtggagacac

16S tick 4 801 taccagcccttgacatcccgatcgcggaaggtggagacac

16S tick 5 801 taccagcccttgacatcccgatcgcggaaggtggagacac

16S tick 6 801 taccagcccttgacatcccgatcgcggaaggtggagacac

16S tick 7 801 taccagcccttgacatcccgatcgcggaaggtggagacac

16S tick 8 801 taccagcccttgacatcccgatcgcggaaggtggagacac

16S tick 9 801 taccagcccttgacatcccgatcgcggaaggtggagacac

16S tick 10 801 taccagcccttgacatcccgatcgcggaaggtggagacac

16S tick 11 801 taccagcccttgacatcccgatcgcggaaggtggagacac

B. henselae 841 cctcctt

16S tick 1 841 cctcctt

16S tick 2 841 cctcctt

16S tick 3 841 cctcctt

16S tick 4 841 cctcctt

16S tick 5 841 cctcctt

16S tick 6 841 cctcctt

16S tick 7 841 cctcctt

16S tick 8 841 cctcctt

16S tick 9 841 cctcctt

16S tick 10 841 cctcctt

16S tick 11 841 cctcctt
