# Supplementary material for: Molecular detection of Bartonella henselae in 11 Ixodes ricinus ticks extracted from a single cat
Source: Parasit Vectors. 2017 Mar 13;10:105. doi: 10.1186/s13071-017-2042-7 (PMC5346845; doi:10.1186/s13071-017-2042-7)
Supplement: Additional file 2: Figure S2. — Alignment 16S-23S-ITS. (DOCX 14 kb) [file 13071_2017_2042_MOESM2_ESM.docx]

**Regier *et al.*, 2017: Molecular detection of *Bartonella henselae* in 11 *Ixodes ricinus* ticks extracted from a single cat**

**Additional file 2: Figure S2.** Alignment 16S-23S-ITS

Reference molecule: B. henselae, strain BM1374165, GenBank: HG969191.1 Region 1483445 to 1483766

B. henselae 1 cgtgaggtcggaggttcaagtcctcccaggcccaccagtt

ITS tick 1 1 cgtgaggtcggaggttcaagtcctcccaggcccaccagtt

ITS tick 2 1 cgtgaggtcggaggttcaagtcctcccaggcccaccagtt

ITS tick 3 1 cgtgaggtcggaggttcaagtcctcccaggcccaccagtt

ITS tick 4 1 cgtgaggtcggaggttcaagtcctcccaggcccaccagtt

ITS tick 5 1 cgtgaggtcggaggttcaagtcctcccaggcccaccagtt

ITS tick 6 1 cgtgaggtcggaggttcaagtcctcccaggcccaccagtt

ITS tick 7 1 cgtgaggtcggaggttcaagtcctcccaggcccaccagtt

ITS tick 8 1 cgtgaggtcggaggttcaagtcctcccaggcccaccagtt

ITS tick 9 1 cgtgaggtcggaggttcaagtcctcccaggcccaccagtt

ITS tick 10 1 cgtgaggtcggaggttcaagtcctcccaggcccaccagtt

ITS tick 11 1 cgtgaggtcggaggttcaagtcctcccaggcccaccagtt

B. henselae 41 tatccattactttcataagtgcttttaaaaaataagtact

ITS tick 1 41 tatccattactttcataagtgcttttaaaaaataagtact

ITS tick 2 41 tatccattactttcataagtgcttttaaaaaataagtact

ITS tick 3 41 tatccattactttcataagtgcttttaaaaaataagtact

ITS tick 4 41 tatccattactttcataagtgcttttaaaaaataagtact

ITS tick 5 41 tatccattactttcataagtgcttttaaaaaataagtact

ITS tick 6 41 tatccattactttcataagtgcttttaaaaaataagtact

ITS tick 7 41 tatccattactttcataagtgcttttaaaaaataagtact

ITS tick 8 41 tatccattactttcataagtgcttttaaaaaataagtact

ITS tick 9 41 tatccattactttcataagtgcttttaaaaaataagtact

ITS tick 10 41 tatccattactttcataagtgcttttaaaaaataagtact

ITS tick 11 41 tatccattactttcataagtgcttttaaaaaataagtact

B. henselae 81 tctaaaaagattgcttctaaaaagcttatcaaaattggca

ITS tick 1 81 tctaaaaagattgcttctaaaaagcttatcaaaattggca

ITS tick 2 81 tctaaaaagattgcttctaaaaagcttatcaaaattggca

ITS tick 3 81 tctaaaaagattgcttctaaaaagcttatcaaaattggca

ITS tick 4 81 tctaaaaagattgcttctaaaaagcttatcaaaattggca

ITS tick 5 81 tctaaaaagattgcttctaaaaagcttatcaaaattggca

ITS tick 6 81 tctaaaaagattgcttctaaaaagcttatcaaaattggca

ITS tick 7 81 tctaaaaagattgcttctaaaaagcttatcaaaattggca

ITS tick 8 81 tctaaaaagattgcttctaaaaagcttatcaaaattggca

ITS tick 9 81 tctaaaaagattgcttctaaaaagcttatcaaaattggca

ITS tick 10 81 tctaaaaagattgcttctaaaaagcttatcaaaattggca

ITS tick 11 81 tctaaaaagattgcttctaaaaagcttatcaaaattggca

B. henselae 121 ggcttattgcttttgtgtgagtaatccaaagttaaagcaa

ITS tick 1 121 ggcttattgcttttgtgtgagtaatccaaagttaaagcaa

ITS tick 2 121 ggcttattgcttttgtgtgagtaatccaaagttaaagcaa

ITS tick 3 121 ggcttattgcttttgtgtgagtaatccaaagttaaagcaa

ITS tick 4 121 ggcttattgcttttgtgtgagtaatccaaagttaaagcaa

ITS tick 5 121 ggcttattgcttttgtgtgagtaatccaaagttaaagcaa

ITS tick 6 121 ggcttattgcttttgtgtgagtaatccaaagttaaagcaa

ITS tick 7 121 ggcttattgcttttgtgtgagtaatccaaagttaaagcaa

ITS tick 8 121 ggcttattgcttttgtgtgagtaatccaaagttaaagcaa

ITS tick 9 121 ggcttattgcttttgtgtgagtaatccaaagttaaagcaa

ITS tick 10 121 ggcttattgcttttgtgtgagtaatccaaagttaaagcaa

ITS tick 11 121 ggcttattgcttttgtgtgagtaatccaaagttaaagcaa

B. henselae 161 attaatggcaaaaaaacagttcaaatgctaaatactaagg

ITS tick 1 161 attaatggcaaaaaaacagttcaaatgctaaatactaagg

ITS tick 2 161 attaatggcaaaaaaacagttcaaatgctaaatactaagg

ITS tick 3 161 attaatggcaaaaaaacagttcaaatgctaaatactaagg

ITS tick 4 161 attaatggcaaaaaaacagttcaaatgctaaatactaagg

ITS tick 5 161 attaatggcaaaaaaacagttcaaatgctaaatactaagg

ITS tick 6 161 attaatggcaaaaaaacagttcaaatgctaaatactaagg

ITS tick 7 161 attaatggcaaaaaaacagttcaaatgctaaatactaagg

ITS tick 8 161 attaatggcaaaaaaacagttcaaatgctaaatactaagg

ITS tick 9 161 attaatggcaaaaaaacagttcaaatgctaaatactaagg

ITS tick 10 161 attaatggcaaaaaaacagttcaaatgctaaatactaagg

ITS tick 11 161 attaatggcaaaaaaacagttcaaatgctaaatactaagg

B. henselae 201 agtcaaaattccttgcaaagtgatttttacagcgtccatt

ITS tick 1 201 agtcaaaattccttgcaaagtgatttttacagcgtccatt

ITS tick 2 201 agtcaaaattccttgcaaagtgatttttacagcgtccatt

ITS tick 3 201 agtcaaaattccttgcaaagtgatttttacagcgtccatt

ITS tick 4 201 agtcaaaattccttgcaaagtgatttttacagcgtccatt

ITS tick 5 201 agtcaaaattccttgcaaagtgatttttacagcgtccatt

ITS tick 6 201 agtcaaaattccttgcaaagtgatttttacagcgtccatt

ITS tick 7 201 agtcaaaattccttgcaaagtgatttttacagcgtccatt

ITS tick 8 201 agtcaaaattccttgcaaagtgatttttacagcgtccatt

ITS tick 9 201 agtcaaaattccttgcaaagtgatttttacagcgtccatt

ITS tick 10 201 agtcaaaattccttgcaaagtgatttttacagcgtccatt

ITS tick 11 201 agtcaaaattccttgcaaagtgatttttacagcgtccatt

B. henselae 241 tggttgatataaattccaaatgctcatagacgtcaatgcc

ITS tick 1 241 tggttgatataaattccaaatgctcatagacgtcaatgcc

ITS tick 2 241 tggttgatataaattccaaatgctcatagacgtcaatgcc

ITS tick 3 241 tggttgatataaattccaaatgctcatagacgtcaatgcc

ITS tick 4 241 tggttgatataaattccaaatgctcatagacgtcaatgcc

ITS tick 5 241 tggttgatataaattccaaatgctcatagacgtcaatgcc

ITS tick 6 241 tggttgatataaattccaaatgctcatagacgtcaatgcc

ITS tick 7 241 tggttgatataaattccaaatgctcatagacgtcaatgcc

ITS tick 8 241 tggttgatataaattccaaatgctcatagacgtcaatgcc

ITS tick 9 241 tggttgatataaattccaaatgctcatagacgtcaatgcc

ITS tick 10 241 tggttgatataaattccaaatgctcatagacgtcaatgcc

ITS tick 11 241 tggttgatataaattccaaatgctcatagacgtcaatgcc

B. henselae 281 tatatgaaactatcggttcaatcatatcgctttgagttat

ITS tick 1 281 tatatgaaactatcggttcaatcatatcgctttgagttat

ITS tick 2 281 tatatgaaactatcggttcaatcatatcgctttgagttat

ITS tick 3 281 tatatgaaactatcggttcaatcatatcgctttgagttat

ITS tick 4 281 tatatgaaactatcggttcaatcatatcgctttgagttat

ITS tick 5 281 tatatgaaactatcggttcaatcatatcgctttgagttat

ITS tick 6 281 tatatgaaactatcggttcaatcatatcgctttgagttat

ITS tick 7 281 tatatgaaactatcggttcaatcatatcgctttgagttat

ITS tick 8 281 tatatgaaactatcggttcaatcatatcgctttgagttat

ITS tick 9 281 tatatgaaactatcggttcaatcatatcgctttgagttat

ITS tick 10 281 tatatgaaactatcggttcaatcatatcgctttgagttat

ITS tick 11 281 tatatgaaactatcggttcaatcatatcgctttgagttat

B. henselae 321 at

ITS tick 1 321 at

ITS tick 2 321 at

ITS tick 3 321 at

ITS tick 4 321 at

ITS tick 5 321 at

ITS tick 6 321 at

ITS tick 7 321 at

ITS tick 8 321 at

ITS tick 9 321 at

ITS tick 10 321 at

ITS tick 11 321 at
